# Effects of High-Resistance Elastic Band Training and a Curcumin-Based Formulation on Neuro-Oxidative and Functional Health in Sedentary Older Adults

**DOI:** 10.3390/healthcare13091055

**Published:** 2025-05-03

**Authors:** Alvaro Juesas, Angel Saez-Berlanga, Javier Gene-Morales, Pablo Jiménez-Martínez, Carlos Alix-Fages, Julio Fernandez-Garrido, Oscar Caballero, Danica Janicijevic, Virginia Zarza, Juan C. Colado

**Affiliations:** 1 Department of Education Sciences, CEU Cardenal Herrera University, 46115 Castellón, Spain; alvaro.juesastorres@uchceu.es; 2 Research Group in Prevention and Health in Exercise and Sport (PHES), University of Valencia, 46010 Valencia, Spain; angel.saez@uv.es (A.S.-B.); p.jimenez@icen.es (P.J.-M.); c.alix@icen.es (C.A.-F.); juan.colado@uv.es (J.C.C.); 3 Department of Physical Education and Sports, University of Valencia, 46010 Valencia, Spain; 4 Department of Health Research, ICEN Research Center, 38002 Santa Cruz de Tenerife, Spain; vzarza@nutris.es; 5 Nursing Department, Faculty of Nursing and Podiatry, University of Valencia, 46010 Valencia, Spain; julio.fernandez@uv.es (J.F.-G.); oscar.caballero@uv.es (O.C.); 6 Faculty of Sports Science, Ningbo University, Ningbo 315211, China; jan.danica@gmail.com; 7 Department of Radiology, Ningbo No. 2 Hospital, Ningbo 315010, China; 8 Department of Sports Sciences and Physical Conditioning, Faculty of Education, Universidad Católica de la Santísima Concepción, Concepción 4090541, Chile

**Keywords:** accentuated eccentric resistance training, maximal strength resistance training, brain-derived neurotrophic factor (BDNF), F2-isoprostanes

## Abstract

**Background/Objectives**: Physical exercise and curcumin supplementation can positively influence parameters related to cognition and neuro-oxidative status. However, research on the combined effects of resistance training with elastic bands and supplementation with a curcumin-based formulation is limited. Moreover, different types of contractions (e.g., concentric and eccentric) may elicit distinct neurophysiological effects. This study evaluated the effectiveness of two high-resistance training (high-RT) programs using elastic bands in improving neuro-oxidative markers (brain-derived neurotrophic factor [BDNF] and F2-isoprostanes), cognitive function, physical performance, and quality of life, and examined the additional benefits of curcumin supplementation. **Methods**: Eighty-one sedentary older adults were randomly assigned to one of the following groups: accentuated eccentric training with either a bio-optimized curcumin formulation (Aecc-Cur) or placebo (Aecc-Pla); maximum strength training with either curcumin (Max-Cur) or placebo (Max-Pla); or a control group receiving curcumin (C-Cur) or placebo (C-Pla) without training. The training groups participated in a 16-week full-body high-RT program using elastic bands. **Results**: BDNF significantly increased in Aecc-Cur and Aecc-Pla (both *p* ≤ 0.020) but showed no changes after Max-Cur or Max-Pla (both *p* ≥ 0.256). All other dependent variables improved similarly across training groups (all *p* ≤ 0.50). Curcumin supplementation combined with exercise significantly reduced F2-isoprostanes in the Max-Cur group compared to Aecc-Pla, and enhanced 6-Minute Walk Test performance in Aecc-Cur and Max-Cur compared to their placebo counterparts. C-Cur showed nonsignificant changes in BDNF, F2-isoprostanes, social functioning, and vitality, while C-Pla worsened these parameters. Notably, at least half of the participants in the experimental groups exhibited clinically significant improvements in 11 of 14 dependent variables. **Conclusions**: Both high-RT protocols led to improvements in almost all dependent variables, with the Aecc program demonstrating greater effectiveness in boosting BDNF, a key neuroprotection marker. Curcumin supplementation alone and with exercise positively influenced neuro-oxidative markers and quality of life.

## 1. Introduction

Being free from illness is a key aspect shared by most definitions of healthy aging [1]. Although a large percentage of people strive to follow healthy lifestyle recommendations, some age-related changes remain inevitable. For instance, aging is the primary risk factor for developing a plethora of neurodegenerative diseases such as Parkinson’s and Alzheimer’s disease [2]. To date, no definitive treatment has been established to prevent or cure their irreversible progression [2]. However, several biomarkers have been strongly linked to neurodegenerative conditions such as Huntington’s, Alzheimer’s, and Parkinson’s diseases, and they hold significant promise as therapeutic targets [3]. One such biomarker is brain-derived neurotrophic factor (BDNF), which has been identified as a crucial regulator of numerous cellular processes essential for maintaining normal brain function [4]. BDNF is also recognized as a potent neuroprotective factor that promotes neurogenesis, synaptogenesis, and dendritogenesis, and also initiates signaling cascades that upregulate the transcription of pro-survival genes in the brain [5]. Oxidative stress markers, such as isoprostanes, are also frequently associated with neurodegenerative diseases, as their serum levels significantly increase in response to free radical-induced tissue damage [6]. The neural tissue of older individuals is particularly vulnerable to oxidative stress due to reduced blood–brain barrier integrity and increased mitochondrial dysfunction [7]. Notably, BDNF and oxidative stress markers are not merely independent indicators of neurodegeneration, as recent studies have shown an inverse relationship between them in various neurodegenerative and mental disorders [8,9]. Therefore, it is not surprising that both BDNF and oxidative stress biomarkers have been identified as key markers of brain health and potential targets for expanding preventive and therapeutic strategies to neurodegenerative diseases.

Regular physical activity and exercise is a promising non-pharmaceutical strategy for preventing and managing neurodegenerative diseases and their associated co-morbid symptoms [9,10,11]. Exercise promotes neurogenesis, increases BDNF and tropomyosin receptor kinase B (a receptor of BDNF), improves synaptic plasticity and memory, and alters long-term potentiation, as well as certain protein concentrations [12]. The neuroprotective effects of physical activity mediated by BDNF are supported by evidence that increases in circulating BDNF originate from brain neural tissue, where it is primarily absorbed and exerts neuroprotective effects [9]. Another mechanism through which physical activity enhances brain health is by modulating oxidative stress and antioxidant capacity [13]. Regular physical activity is generally associated with reduced oxidative stress, which helps maintain the structural integrity of cellular components, including membranes, proteins, lipoproteins, enzymes, hormones, and genetic material [14]. In neural tissue, reduced oxidative stress lowers the prevalence of pathological aggregation of misfolded proteins, such as the extracellular amyloid plaques and intraneuronal neurofibrillary tangles associated with Alzheimer’s disease, as well as the intracellular Lewy bodies observed in Parkinson’s disease [15]. Additionally, regular physical activity has been hypothesized to mitigate cognitive decline through modulation of the factors participating in the crosstalk between skeletal muscle and the brain, such as neurotrophins and oxidative stress parameters [16]. Moreover, several studies have reported positive effects of regular physical activity in patients suffering from depression [17,18].

Not all types of exercise and training intensities are equally effective in improving brain health [19]. Surprisingly, low-intensity (aerobic and resistance) training programs, typically recommended for older adults, are less effective than high-intensity training protocols in boosting BDNF levels [20]. Aerobic exercise positively affects brain health by stimulating neurogenesis, angiogenesis, and synaptogenesis, which are regulated by BDNF and growth factors such as insulin-like growth factor 1 (IGF-1) and can change brain function and structure, e.g., an increase in brain volume [21]. Muscle contractions within resistance training (RT) increase mitochondrial function, neurochemical markers of brain neuronal density, and enhance neurotransmission, including BDNF and IGF-1, and decrease homocysteine levels [22]. This triggers complex neurobiological processes, e.g., brain plasticity and angio-neurogenesis, that lead to functional and structural brain changes that stimulate cognitive function [22]. Additionally, combined aerobic and RT, e.g., multicomponent training, has shown positive results for global cognition in people with cognitive impairment through shared mechanisms between aerobic and RT [23]. To our knowledge, only two studies have evaluated the effects of physical exercise on BDNF in older participants, both showing that low- to moderate-intensity walking endurance programs can elevate BDNF post-intervention [24,25]. Therefore, it would be valuable to investigate whether high-intensity RT could produce chronic adaptations in BDNF levels in older adults. Additionally, different types of muscle contractions (i.e., eccentric vs. concentric) may modulate neurophysiological responses through stretch or shear stresses, which promote mechanosensing-induced signaling pathways and differ in lactate production and muscle damage [26]. However, no previous studies have compared the effects of accentuated eccentric (Aecc) and maximal strength training (Max) on neurotrophin release in older adults.

When it comes to understanding the effects of physical exercise on oxidative stress, current knowledge is relatively well-established. Acute bouts of exercise are known to temporarily induce oxidative stress, particularly in older adults, due to the age-related decline in the efficiency of their endogenous antioxidant systems [27]. However, repeated episodes of exercise-induced oxidative stress appear to enhance the body’s capacity to manage oxidative stress by upregulating antioxidant enzyme activity [28,29]. This adaptive response, which involves the activation of signaling pathways that boost antioxidant defenses, seems to be possible at any stage of life, even in older adults. For instance, physically fitter older individuals have been shown to exhibit lower oxidative stress levels in response to various oxidative challenges compared to their less active peers [28,29]. Although several studies have demonstrated that the positive effects of training programs can be achieved through RT routines [30,31], these benefits are typically associated with low-to-moderate aerobic training protocols [32,33]. Therefore, it would be of considerable interest to investigate whether similar improvements in oxidative stress markers can be obtained through high-RT programs in older adults, considering the numerous additional benefits of RT for this population [34].

Besides physical activity, nutritional supplements are regarded as powerful tools to enhance health and overall well-being [35,36]. Among them, curcumin supplementation has emerged as a promising option, particularly in the context of neural health, cognitive decline, and symptoms of depression [37,38]. Curcumin has a pleiotropic activity within cells and interacts with many proteins [39]. Additionally, it decreases oxidative damage in the brain; and regulates BDNF, malondialdehyde and superoxide dismutase activities, and extracellular signal-regulated kinase, a molecule that regulates a variety of cellular functions, including survival [12,35,36]. The observed reduction in oxidative stress with curcumin is often accompanied by increased BDNF levels, reinforcing the hypothesis of an inverse relationship between oxidative stress and BDNF concentration [35,40]. Notably, the positive effects of curcumin supplementation appear to be further enhanced by regular physical activity [35]. In addition, curcumin has been shown to slow cognitive decline in older adults [37] and enhance the efficacy of antidepressant treatments in depressive disorders [38]. Given these findings, we aimed to explore whether curcumin supplementation can further amplify the effects of high RT on a cluster of brain-health-related variables in healthy older adults.

To address the identified research gaps, this study aimed (I) to compare the effectiveness of two high-RT protocols (Aecc and traditional concentric-eccentric Max) in improving neuro-oxidative markers (BDNF and F2-isoprostanes), cognitive functioning, physical performance, and health-related quality of life, as well as (II) to investigate whether combining high-RT programs with curcumin supplementation can further enhance these improvements. We hypothesized that both high-RT programs would effectively improve all dependent variables and that these improvements would be further enhanced by curcumin supplementation. However, no specific hypothesis was formed regarding which of the two protocols (Aecc or Max) would be more effective, due to the absence of relevant comparative studies for this population. The findings of this study may help determine the most suitable combination of high-RT regimen and supplementation for older adults to mitigate age-related neural and physical-cognitive decline, while improving overall quality of life.

## 2. Materials and Methods

### 2.1. Participants

An a priori analysis conducted using G*Power (Version 3.1.9.3) determined that a total of 60 participants were needed for a two-way ANOVA, with six groups and a pre-post measurement design. This ensured 80% statistical power, an effect size of 0.25, a significance level of 0.05, and accounted for a correlation of 0.5 between measurements. To account for potential dropouts caused by the study duration and participant characteristics, we recruited 92 participants of both sexes, 81 of whom successfully completed the study (age: 68.17 ± 4.60 years, height: 164.86 ± 6.77 cm, body weight: 71.29 ± 11.31 kg, weekly physical activity: 756.19 ± 9.88 metabolic equivalent of task [METs]). The 11 dropouts were due to compliance issues, with no cases attributed to injuries, adverse effects from exercise, or reactions to supplementation. Participants were randomly allocated into six groups: accentuated eccentric training with curcumin supplementation (Aecc-Cur, n = 16) or placebo (Aecc-Pla, n = 13), maximum strength training with curcumin (Max-Cur, n = 10) or placebo (Max-Pla, n = 10), and a control group receiving either curcumin (C-Cur, n = 15) or placebo (C-Pla, n = 17). Although both researchers and participants were aware of the training groups due to their active involvement in the exercises, they were blind to whether the supplementation capsules contained curcumin or a placebo.

To qualify for inclusion in the study, participants had to meet several eligibility criteria: (i) be a sedentary adult aged 60 or older, (ii) maintain functional independence without the need for walking aids or stair assistance, (iii) pass a medical examination confirming their ability to safely participate in a RT program, (iv) be a non-smoker and not consume alcohol regularly, and (v) have refrained from consuming antioxidant supplements (e.g., vitamins C, E, A, omega-3) for at least six weeks prior to the study’s start. Participants with any disease or condition that could compromise their safe participation in the RT program were excluded from the study. These included severe visual or hearing impairments, terminal illnesses, and cognitive impairment (i.e., a score below 23 on the Mini-Mental State Examination test). Additionally, individuals were excluded if they were taking or had previously taken medications potentially interfering with the variables measured or if they had experienced significant body weight fluctuations (≥10% change within the past year). The study’s purpose, procedures, potential health benefits, and possible discomforts were thoroughly explained to all participants before the study commenced. Subsequently, informed consent was obtained from each participant. Participants were informed that they were free to withdraw from the study at any time. This research is contained within a larger project that aims to evaluate how physical exercise in combination with nutraceuticals affects older adults’ functionality and health. The study complied with the principles of the Declaration of Helsinki, was approved by the University of Valencia Ethics Committee (IRB: 1861154), and was registered on ClinicalTrials.gov (NCT06620666), date: 30 September 2024.

### 2.2. Study Design

**Figure 1:**
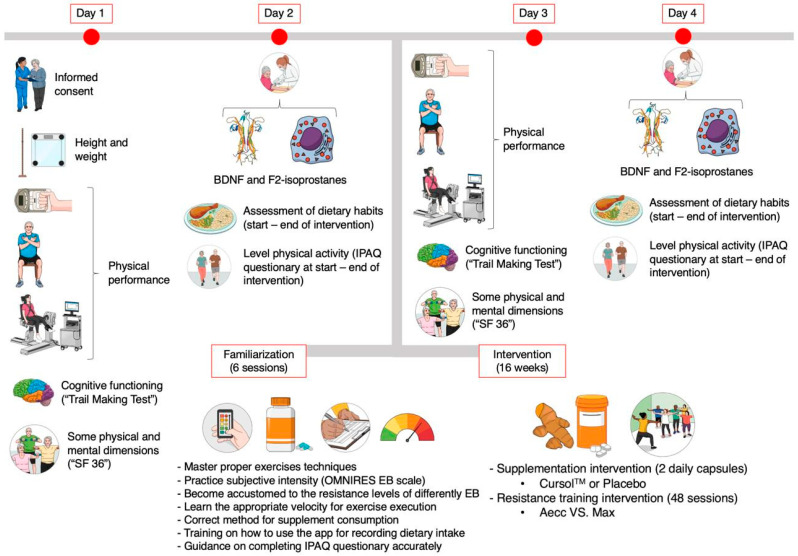
Study design. BDNF, brain-derived neurotrophic factor; EB, elastic bands; IPAQ, International Physical Activity Questionnaire; SF 36, 36-Item Short Form Health Survey. Created with Mindthegraph.com (accessed on 24 February 2025).

**Figure 2:**
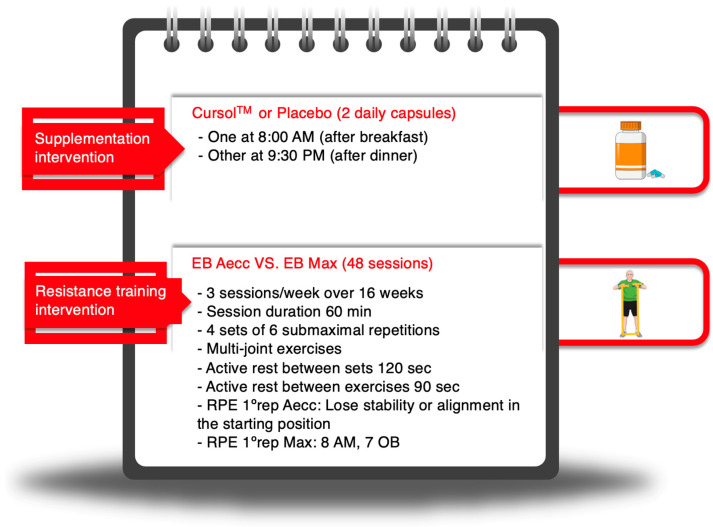
Experimental protocols for supplementation and training intervention. Aecc, accentuated eccentric training; EB, elastic bands; Max, maximum strength training; RPE 1° rep, rate of perceived exertion of the first repetition; AM, active muscles; OB, overall body; Min, minutes; Sec, seconds. Created with Mindthegraph.com (accessed on 17 April 2025).

A longitudinal, triple-blinded pre-post study was conducted to evaluate sedentary older adults’ long-term adaptations in neuroprotection, oxidative stress, physical performance, cognition, and health-related quality of life parameters with two high-RT programs (Aecc vs. Max) combined with curcumin supplementation. The study groups that performed exercise completed pre-intervention testing; a familiarization period of two weeks (6 sessions); and 16 weeks (48 sessions) of RT either Aecc-Cur, Aecc-Pla, Max-Cur, or Max-Pla, and post-intervention measurements. Three training sessions of approximately 1 h were conducted each week on alternating days, to ensure a minimum rest period of 48 h between sessions. The control groups (C-Cur and C-Pla) only took part in the initial and final assessments, without engaging in familiarization or RT sessions. The initial and final testing were identical, consisting of measurements of neuroprotective parameters (BDNF) and oxidative stress markers (F2-isoprostanes); cognitive assessment (Trail Making Test A and B); physical performance evaluations (30-Second Chair Stand, Timed Up-And-Go Test, Six-Minute Walk Test, manual dynamometry, and isokinetic leg strength); and health-related quality of life assessment, including general health, social functioning, vitality, and mental health dimensions from the SF-36 questionnaire (see Figure 1 and Figure 2 for experimental setup). All testing and training sessions were carried out at approximately the same time of day under consistent environmental conditions to ensure standardization.

### 2.3. Testing Procedures

All the measurements, both at the pre- and post-intervention, took place on two different days at the University of Valencia. One day was used for anthropometry, performance, functionality, and cognitive assessments at the Faculty of Physical Activity and Sports Sciences, with the other day for blood extraction at the Faculty of Nursing. Pre-intervention measurements were conducted before starting the familiarization period and final evaluations took place 48 h after the completion of the intervention.

For day one, each participant attended the laboratory in the morning (between 8:30 and 10:00 a.m.). Upon arrival, they remained seated for five minutes to stabilize baseline conditions. Subsequently, height was measured using a portable stadiometer (Seca GmbH & Co. KG, Hamburg, Germany), and body weight was measured using an electronic bascule (Seca 878 model; Seca GmbH & Co. KG, Hamburg, Germany). To ensure measurement accuracy, participants wore light clothing and refrained from caffeine consumption and exercise for at least 8 and 24 h, respectively. The characteristics of the tests performed on the first day of initial and final testing were as follows:

*Cognitive assessment:* The Trail Making Test (TMT) A and B is a neuropsychological test used to assess key cognitive functions such as executive functioning, speed of processing, mental flexibility, scanning, and visual search [41]. In TMT-A, participants were instructed to connect numbers sequentially in ascending order (1 → 2 → 3, etc.). In TMT-B, they alternated between numbers and letters in ascending order (1 → A → 2 → B, etc.). For both test conditions, completion time served as the primary outcome measure.

*Health-related quality of life assessment:* According to our study design, four key dimensions of the Short Form Health Survey SF 36 (general health, social functioning, vitality, and mental health) were selected to assess participants’ health status. Each dimension was evaluated by coding, aggregating, and transforming responses into a 0 to 100 scale, where 0 represents the poorest health state and 100 is the best. Since the SF 36 is not designed to produce an overall score, a separate score was generated for each dimension [42]. Previous research has demonstrated the reliability and effectiveness of the SF 36 in assessing health-related quality of life in older adults [43].

*Physical performance:* This was assessed using five tests: (I) maximal grip strength, following the protocol suggested by Roberts et al. [44]; (II) isokinetic strength of knee flexors and extensors in the dominant leg, measured using an isokinetic dynamometer (Biodex Medical™, Shirley, NY, USA) that included the analysis package Advantage (version 3.2, Biodex System Advantage, Shirley, NY, USA). The test was conducted within a range of motion from 5° to 90° at an angular velocity of 60°/s, with participants performing five consecutive flexion and extension movements; (III) 30 s Chair Stand Test, evaluating local muscular endurance; (IV) 6-Minute Walk Test, assessing aerobic capacity; and (V) Timed Up and Go Test, measuring proactive balance and agility. The last three tests were selected from the Senior Fitness Test Battery [45], where they are thoroughly described.

On the second day of testing, participants, after fasting for at least 8 h, visited the Faculty of Nursing, where trained nurses collected blood samples. The samples were stored in SST tubes (Greiner Bio-one GmbH, Kremsmünster, Austria), designed to separate serum from blood cells during centrifugation. They were then centrifuged (MIXTASEL-BL, J.P. Selecta S.A., Barcelona, Spain) at 3000 rpm for 15 min at 4 °C, after which the serum samples were preserved at −80 °C until further analysis. The levels of 8-isoprostane PGF2α, a specific type of F2-isoprostanes, were quantified using enzyme-linked immunosorbent assays (ELISA). The measurements were performed with an EIA-3080 kit, which offers a sensitivity of approximately 29 pg/mL and a detection range from 1 to 10,000 pg/mL. The intra-assay variability for this kit was less than 11.00%, while the inter-assay variability was also under 11.00%, ensuring reliable and reproducible results. BDNF was measured using an EIA-5968 kit, which has a sensitivity of approximately 1.11 pg/mL and a detection range between 0 and 500 pg/mL. The intra-assay variability for the BDNF kit was less than 9.00%, and the inter-assay variability was less than 8.50%, further indicating high accuracy and consistency in the measurements. Both kits were supplied by DRG Instruments GmbH (Marburg, Germany) and analysis were conducted with Labtec ELISA Microplate Reader LT-5000ms Lab (Labtec International LTD, East Sussex, UK). All procedures adhered to Clinical and Laboratory Standards Institute (CLSI) guidelines.

The familiarization sessions that took place after the pre-intervention measurements were used to teach participants the execution of each exercise, to adjust resistance levels corresponding to the color-coded bands, and to optimize movement speed and repetition frequency.

### 2.4. Training Procedures

The experimental groups completed a total of 48 sessions, held three times per week over a 16-week intervention period. Each session followed the guidelines of the American College of Sports Medicine [46] and included 5–10 min of general warm-up, 40–50 min of the main workout, and 5–10 min of flexibility and relaxation exercises. During the main workout, participants performed four sets of six submaximal repetitions of multi-joint exercises, including lunges, standing horizontal chest presses, standing horizontal hip hinges, and standing horizontal rows, all executed using CLX elastic bands (TheraBand^®^; Hygenic Corporation, Akron, OH, USA) [46]. The program was designed to target both major and minor muscle groups efficiently. Rest intervals were set at 2 min between sets and 90 s between exercises. Active rests were applied to enhance motivation and adherence; these included light-intensity coordination and simple cognitive exercises, including basic dancing, reaction-time drills, and exercises involving response to pre-learned stimuli. A summary of the training procedures can be found in Figure 2.

All multi-joint exercises during the main phase incorporated elastic bands attached to a wall-mounted support, with participants holding the ends of the bands. The resistance was adjusted both objectively (by using bands of different tension levels, combining multiple bands, or altering the starting position relative to the wall) and subjectively (based on participants’ rate of perceived exertion [RPE] of the first repetition, using the OMNI-RES scale for elastic bands [47]). Once the optimal resistance had been determined during the first set, a floor marker was placed to ensure consistent resistance across the remaining sets. Exercise pace was controlled with a metronome [47] and supervised by an experienced sports scientist. Following the manufacturer’s safety guidelines, elastic bands were stretched to a maximum of 300% of their resting length to prevent breakage. Once this limit was reached, the band was replaced with one offering greater viscoelastic stiffness to maintain higher resistance levels. Both high-RT programs were designed according to the guidelines for older adults [48], with the specific features of each program detailed as follows:

*Accentuated eccentric elastic band program (Aecc):* Participants began each exercise in an initial position close to the wall, completing the concentric phase with no tension in the elastic bands (e.g., arms extended in front of the body at shoulder width). Once the concentric phase was completed, they gradually moved away from the wall while maintaining the same joint alignment. When they could no longer maintain stability or proper positioning (e.g., elbows began to flex), a reference marker was placed on the floor to indicate that the optimal resistance had been reached. Once the optimal resistance was established, participants proceeded with the eccentric phase of the movement (e.g., flexing their elbows in a controlled manner over 5 s). After completing the controlled eccentric phase, they returned to the initial position within 2 s and began the next repetition. This method ensured that participants consistently performed the exercises at 100% of their maximal capacity, typically reaching an RPE of 7 or 8 by the end of the first repetition of each set.

*Maximal strength elastic band program (Max):* Participants walked away from the wall until they reached an elastic band tension that resulted in an RPE of 7 in the active muscles or 8 in the overall body by the end of the first repetition of each set [47,49]. Once the optimal tension was reached, a floor marker was placed to ensure consistency across all repetitions and sets. Standing at this point, participants executed both phases of each exercise, i.e., concentric and eccentric, with each phase lasting 2 s. After completing all repetitions in a set, they returned to the wall to rest before starting the next set. A sports scientist closely monitored each participant to ensure they consistently maintained the prescribed RPE, making sure they never reached 10.

### 2.5. Procedures for Monitoring Supplementation, Diet, and Physical Activity

Participants from both the experimental and control groups were randomly assigned to receive either curcumin or placebo. Each participant was instructed to take two capsules per day, one after breakfast (approximately 8:00 a.m.) and one after dinner (approximately 9:30 p.m.). Capsules were composed of 250 mg of Cursol™, a bio-optimized curcumin formulation with dual patents that ensure the preservation of its bio-active properties (Nutris We Care About You, Madrid, Spain). Cursol™ is standardized to 2.1% (5.25 mg) curcumin; complying with recommended dosage for human consumption and older populations [50,51,52]. In detail, each Cursol™ capsule contained dibasic calcium phosphate anhydrous (E341, stabilizer), Polysorbate 80 (E433, emulsifier), turmeric rhizome extract (*Curcuma longa* L.), and citric acid (E330, acidity regulator).

The placebo capsule contained 250 mg of maltodextrin and identical excipients, ensuring it was indistinguishable in appearance from the curcumin capsule. Thus, participants consumed a total of 500 mg daily, either as Cursol™ or placebo. Blinding was rigorously maintained for both participants and investigators throughout the study.

All participants were instructed to maintain their regular dietary routines throughout the study. Dietary intake was monitored at both the beginning and the end of the study over three non-consecutive days (two weekdays and one weekend day) using the validated smartphone app MyFitnessPal version 25.13.0-40874 (MyFitnessPal LLC, San Francisco, CA, USA) [13,14]. Proper use of the app and accurate recording of dietary intake were supervised by an experienced nutritionist to ensure compliance and reliability.

Participants answered pre- and post-intervention the International Physical Activity Questionnaire (IPAQ), a self-reported tool that calculates the METs derived from the physical activity. Participants were instructed to evaluate their physical activity levels independently of their involvement in the study’s exercise sessions.

### 2.6. Statistical Analyses

Descriptive data are reported as means and standard deviations (SD). The Shapiro–Wilk test confirmed that BDNF, TMT-A, TMT-B, and the vitality dimension were the only variables normally distributed. The changes from pre- to post-intervention were calculated as delta percentage using the formula %Δ = [(post-intervention score − pre-intervention score)/pre-intervention score] × 100). A two-way analysis of variance (ANOVA) of repeated measures assessed the influence of time (pre-test vs. post-test) as the within-participant factor and experimental group (Aecc-Cur vs. Aecc-Pla vs. Max-Cur vs. Max-Pla vs. C-Cur vs. C-Pla) as the between-participant factor on the variables that were normally distributed and showed no between-group differences at pre-intervention (i.e., BDNF, TMT-A, TMT-B, and vitality). For normally distributed variables not equalized at baseline (BDNF), we applied a two-way analysis of covariance (ANCOVA), including the pre-intervention values as covariates [15,16]. We used 95% confidence intervals to conduct within- and between-group comparisons. Post hoc comparisons were conducted using a Least Significant Difference (LSD) test, with effect sizes (ES) calculated as partial eta squared (ƞp^2^).

The Kruskal–Wallis test was used to analyze differences between experimental groups at both pre- and post-test time points for variables that did not follow a normal distribution. These variables included the 30 s Chair Stand Test, Timed Up-and-Go Test, 6-minute Walk Test, maximal grip strength, knee extensor strength, knee flexor strength, F2-isoprostanes, general health, social functioning, and mental health. We used Quade’s rank analysis of covariance (Quade’s ANCOVA), including the pre-intervention values of the 6-Minute Walk Test and social functioning as covariates to adjust for pre-existing differences [53,54]. When a test statistic was significant, we used 95% confidence intervals to explore significant within- and between-group differences. Friedman’s test was used to evaluate the influence of time. The ES was calculated using Kendall’s Coefficient of Concordance (*w*) and interpreted following Cohen’s guidelines [55]. Finally, we evaluated post hoc differences by means of Wilcoxon and Mann–Whitney U tests for comparisons within and between groups; the ES was reported as per Hedge’s g.

Spearman’s rank-order correlation was used to explore the correlations between the changes in the dependent variables following the intervention, calculated as the difference between post-intervention and pre-intervention values. The minimal clinically important difference (MCID) endpoints and cutoff points were extracted from previous literature similar in terms of intervention, outcome measure, and population [56]. The MCID is defined as the smallest change in a variable that is considered meaningful and beneficial, justifying a treatment change, without causing significant side effects or high costs [57,58]. MCID endpoints refer to specific levels of improvement associated with positive clinical effects while cutoff points indicate thresholds to identify risks or classify participants. With these values in mind, we manually calculated the percentage of participants that achieved each endpoint/cutoff point.

The intention-to-treat approach was employed, wherein baseline measurements for participants who withdrew from the study were carried forward to the post-intervention phase to address missing data [23]. The qualitative interpretation of partial eta squared (ƞp^2^) values was as follows: 0.01–0.06 indicated a small effect, 0.06–0.14 a medium effect, and values greater than 0.14 represented a large effect. The ES was reported as Hedge’s *g*, aiming to compensate for potential sample size biases; results were categorized as trivial (<0.20), small (0.20–0.49), moderate (0.50–0.80), and large (>0.80) [55]. Finally, correlations were qualitatively interpreted as 0.00–0.10 (negligible), 0.10–0.39 (weak), 0.40–0.69 (moderate), 0.70–0.89 (strong), and 0.90–1.00 (very strong) [59]. Statistical analyses were performed using IBM SPSS Statistics software (version 28.0.1.1, IBM Corp., Armonk, NY, USA). The alpha level for statistical significance was set at *p* < 0.05.

## 3. Results

**Figure 3:**
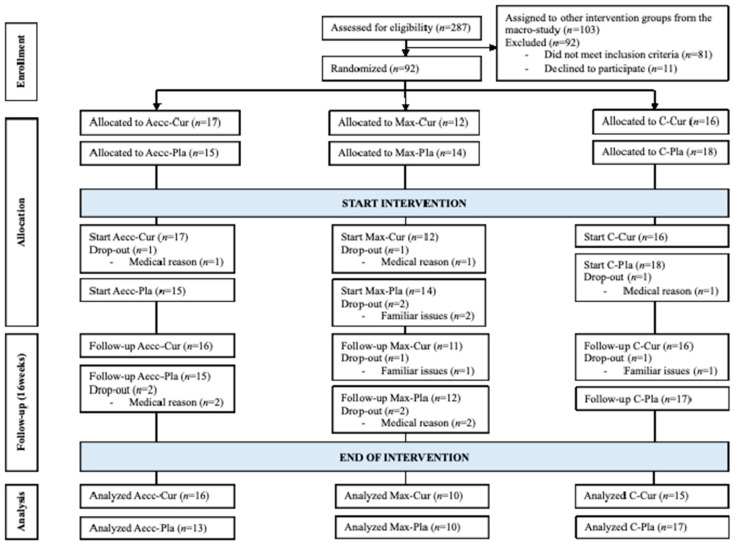
Participants flow throughout the study.

Adherence to the training program exceeded 85% across all training groups, while self-reported adherence to the supplementation protocol was 89.39%. No significant differences were observed at baseline in age, height, weight, weekly physical activity, or dietary intake (total calories, protein, carbohydrates, and lipids) between groups (all *p* > 0.05). Similarly, no differences were found in physical activity levels between experimental groups throughout the study (*p* = 0.152). Figure 3 presents the participants flow throughout the study.

### 3.1. Neurogenesis and Oxidative Stress Parameters

A significant time × group interaction was observed for BDNF (F = 18.25, *p* < 0.001, ηp^2^ = 0.20), indicating differing trends between the experimental and control groups. Specifically, BDNF levels increased in the experimental groups following the RT programs, whereas they decreased in the control groups. The main effect of time was also significant for BDNF (F = 19.35, *p* < 0.001, ηp^2^ = 0.21). Pre-post comparisons revealed that Aecc groups showed significant improvements in BDNF levels after the intervention, whereas the Max group did not show a significant change. A significant time × group interaction was also found for F2-isoprostane (H = 38.46, *p* < 0.001, ηp^2^ = 0.48), indicating lower concentrations in the experimental groups compared to the control groups. The main effect of time was significant for F2-isoprostane (χ^2^ = 5.44, *p* = 0.020, W = 0.10), with significant reductions observed in all experimental groups. Detailed pairwise comparisons are presented in Table 1.

### 3.2. Cognitive Functioning

A significant time × group interaction was observed for both TMT-A (F = 76.50, *p* < 0.001, ηp^2^ = 0.51) and TMT-B (F = 47.76, *p* < 0.001, ηp^2^ = 0.39), indicating superior executive functioning, processing speed, mental flexibility, visual scanning, and search abilities in the experimental groups compared to the control groups. Additionally, a significant main effect of time was found for both TMT-A (F = 12.09, *p* < 0.001, ηp^2^ = 0.45) and TMT-B (F = 3.85, *p* = 0.004, ηp^2^ = 0.20), reflecting overall improvement from baseline to the 16-week follow-up. Detailed pairwise comparisons are presented in Table 2.

### 3.3. Health-Related Quality of Life Dimensions

The interaction time × group was significant in all dimensions (general health: H = 36.29, *p* < 0.001, ηp^2^ = 0.45; social functioning: H = 29.81, *p* < 0.001, ηp^2^ = 0.37; vitality: F = 6.94, *p* < 0.001, ηp^2^ = 0.32; mental health: H = 17.00, *p* = 0.004, ηp^2^ = 0.21). A significant effect of time was also found for all dimensions (general health: χ^2^ = 35.53, *p* < 0.001, W = 1.78, social functioning χ^2^ = 8.17, *p* = 0.004, W = 1.59, vitality: F = 88.99, *p* < 0.001, W = 0.55, and mental health: χ^2^ = 17.82, *p* < 0.001, W = 1.67). Although not significant, percent improvements and effect sizes were consistently larger in the experimental groups receiving curcumin supplementation compared to the placebo groups within the same training modality (median ES of 0.87 for curcumin groups vs. 0.71 for placebo groups). Similarly, curcumin supplementation helped preserve scores across all health-related quality of life parameters in the control group, whereas the placebo group experienced declines. These differences reached statistical significance for general health (*p* = 0.050) and social functioning (*p* = 0.049) scores. Pairwise comparisons are presented in Table 3.

**Figure 4:**
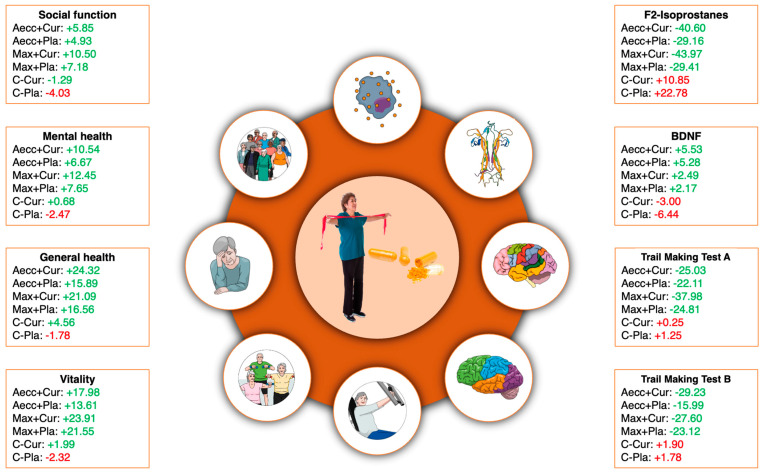
Percentage changes in neuro-oxidative markers, cognitive functioning, and quality of life variables. Green indicates positive effects for the study participants. For a more comprehensive statistical interpretation of this figure, the reader is referred to the tables provided in Results Section.

Figure 4 summarizes neuro-oxidative markers, cognitive functioning, and quality of life outcomes.

### 3.4. Physical Performance

A significant time × group interaction was observed for all physical performance parameters, indicating greater improvements in the experimental groups compared to the control groups, whose performance either remained stable or slightly declined. The significant interactions were as follows: 30-Second Chair Stand (H = 29.68, *p* < 0.001, ηp^2^ = 0.36), Timed Up and Go (H = 20.06, *p* = 0.001, ηp^2^ = 0.25), 6-Minute Walk Test (H = 27.09, *p* < 0.001, ηp^2^ = 0.34), maximal grip strength (H = 12.92, *p* = 0.024, ηp^2^ = 0.16), leg flexion strength (H = 36.77, *p* < 0.001, ηp^2^ = 0.46), and leg extension strength (H = 15.63, *p* = 0.008, ηp^2^ = 0.20). A significant main effect of time was also found for all physical performance parameters, reflecting an overall improvement from baseline to the end of the 16-week intervention: 30 s Chair Stand (χ^2^ = 20.90, *p* < 0.001, W = 1.72), Timed Up and Go (χ^2^ = 16.61, *p* < 0.001, W = 1.28), 6-Minute Walk Test (χ^2^ = 7.58, *p* = 0.006, W = 1.65), maximal grip strength (χ^2^ = 12.18, *p* < 0.001, W = 1.68), leg flexion strength (χ^2^ = 15.51, *p* < 0.001, W = 0.19), and leg extension strength (χ^2^ = 21.28, *p* < 0.001, W = 0.26). Although not significant, percent improvements and effect sizes were consistently larger in the experimental groups receiving curcumin supplementation compared to placebo groups within the same training modality (median ES of 1.37 for curcumin groups vs. 0.81 for placebo groups). Detailed pairwise comparisons are presented in Table 4.

**Figure 5:**
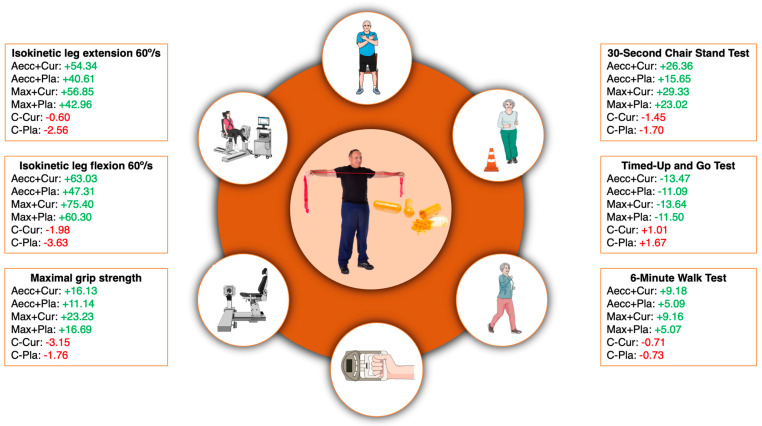
Percentage changes in physical performance variables. Green indicates positive effects for the study participants. For a more comprehensive statistical interpretation of this figure, the reader is referred to the tables provided in Results Section.

Figure 5 summarizes physical performance outcomes.

### 3.5. Bivariate Correlation Analysis (Spearman’s ρ)

**Figure 6:**
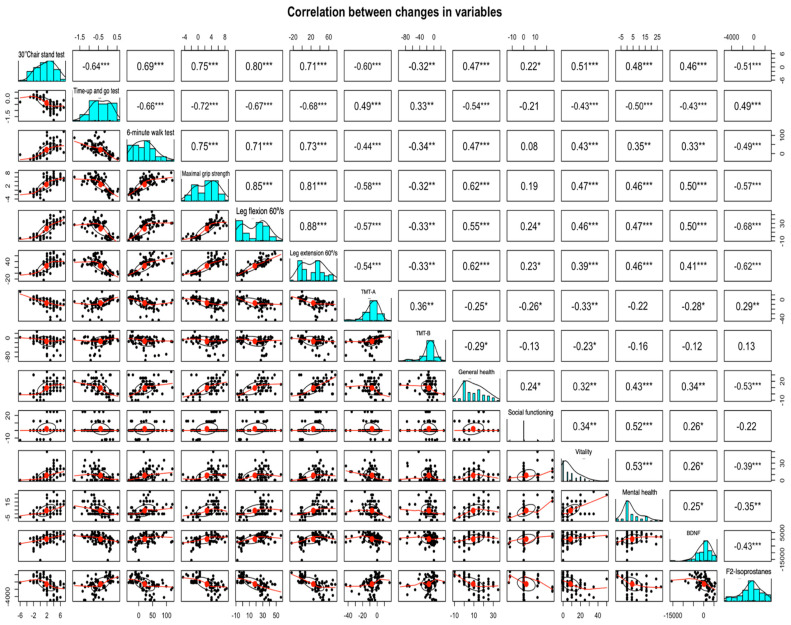
Correlation between changes in dependent variables and pre-intervention and post-intervention BDNF, brain-derived neurotrophic factor; histograms presented in a diagonal line show the distribution of each variable, the lower triangle below the histogram diagonal shows bivariate scatter plots including a fitted line, the upper triangle above the histogram diagonal shows the correlation coefficients and identifies statistical significance as (***) correlation is significant at the *p* = 0.000 level (2-tailed); (**) correlation is significant at the *p* < 0.001 level (2-tailed); (*) correlation is significant at the *p* = 0.005 level (2-tailed).

The correlational analysis revealed 81 out of 91 significant correlations between the changes in the dependent variables during the intervention (Figure 6). BDNF and F2-isoprostanes were significantly associated with changes in all dependent variables, except for BDNF with TMT-B (*ρ* = 0.16) and F2-isoprostanes with social functioning (*ρ* = −0.19). Generally, moderate and significant correlations were obtained between BDNF and enhancements in all physical performance variables (0.39 ≤ *ρ* ≤ 0.50, all *p* < 0.001), TMT-A (*ρ* = −0.42, all *p* < 0.001), and general health dimension (*ρ* = −0.44, *p* < 0.001). In parallel, decrements in oxidative stress were moderately and significantly correlated with all physical performance variables (−0.56 ≤ *ρ* ≤ −0.69, all *p* < 0.001), TMT-A (*ρ* ≤ −0.45, *p* < 0.001), and all health-related quality of life variables, except for cognitive functioning (−0.46 ≤ *ρ* ≤ −0.65, all *p* < 0.001). Importantly, a moderate, and significant correlation was obtained between BDNF and F2-isoprostanes (*ρ =* −0.54, *p* < 0.001).

### 3.6. Clinical Relevance

At least half of the participants in the four experimental groups (Aecc or Max combined with supplement or placebo) achieved endpoints or cutoff points in at least 11 out of the 14 dependent variables, indicating benefits in reducing physical impairment, neurodegenerative and cardiovascular diseases, and mortality (Table 5).

## 4. Discussion

This study aimed to investigate the effectiveness of two high-RT elastic band protocols (Aecc vs. Max) in improving neuro-oxidative markers, cognitive function, physical performance, and quality of life, while also examining whether combining high-RT programs with curcumin supplementation could enhance these benefits. The main findings revealed that BDNF levels only increased after the Aecc protocol. All other variables such as F2-isoprostanes, cognitive functioning, all physical performance variables, and all dimensions of health-related quality of life (except for social functioning in the Max-placebo group) improved comparably after both high-RT protocols. Furthermore, curcumin supplementation contributed to a greater reduction in F2-isoprostanes in the Max group compared to the Aecc-Pla and enhanced 6-Minute Walk Test results in both high-RT protocols compared to the respective placebo groups. In the control groups, it prevented BDNF reduction, limited the increase in F2-isoprostanes levels, and enhanced perceptions of social functioning and vitality compared to the respective placebo control groups. Additionally, significant moderate correlations were observed between neuro-oxidative markers and cognitive function, physical performance, and health-related quality of life parameters. These correlations may be explained by elevated levels of BDNF and reduced oxidative stress, improving synaptic plasticity and mitochondrial efficiency, reducing inflammation and central fatigue pathways, thus contributing to increased cognition, neuromuscular performance, and quality of life [7]. Importantly, at least half of the participants in the four experimental groups achieved significant clinical improvements in 11 out of 14 dependent variables. Overall, these findings suggest that while both high-RT protocols were effective in improving performance across all dependent variables, the Aecc protocol demonstrated an advantage by significantly increasing BDNF levels—a critical neuroprotective marker, particularly in older populations. Additionally, curcumin supplementation proved to be a valuable adjunct for supporting neuroprotection, decreasing oxidative stress, improving physical performance, and enhancing perceived well-being.

In our study, the Aecc protocol, not the Max-RT protocol, was the only intervention that significantly increased BDNF levels. This finding partially contradicts previous studies, which reported that high-RT is effective for improving BDNF levels, regardless of intervention duration (6–26 weeks), with the effects being more pronounced in older adults with poor health [71]. One possible explanation for the differing effectiveness of the two high-RT protocols in our study could lie in the variation in time under tension between the different high-RT programs. For instance, participants in the Aecc group performed a controlled eccentric action lasting 5 s per repetition, with the concentric phase executed without elastic band resistance. In contrast, participants in the Max group performed both concentric and eccentric actions with resistance, each lasting 2 s. The prolonged eccentric phase in the Aecc protocol may have induced greater lactate production, which is increasingly recognized as a key mediator linking high-RT exercise programs with higher BDNF expression [72]. Specifically, previous studies have proposed that lactate produced by working muscles is released into the bloodstream, crosses the blood–brain barrier, and is taken up by astrocytes, which then shuttle it to neurons [73]. Once in neurons, lactate acts as a metabolic and signaling molecule that triggers BDNF expression [73]. However, it remains unclear whether repeated lactate peaks during chronic RT protocols served as the underlying mechanism for the long-term BDNF improvement observed in this study, as the role of lactate in this context has been primarily investigated during acute bouts of exercise. It is worth noting that all participants belonging in the experimental groups exceeded clinically relevant changes when it comes to BDNF. This is especially relevant in neurophysiological terms, as BDNF is related to neuroprotection through neurogenesis, synaptogenesis, and dendritogenesis, and initiates signaling cascades that upregulate transcription of pro-survival genes in the brain [5].

Both high-RT programs were effective in improving F2-isoprostanes, a compound considered as one of the most important oxidative stress markers. This finding is particularly important, as previous studies have predominantly recommended moderate-intensity aerobic exercise to enhance the body’s antioxidative capacity [74], while expressing concerns about high-RT programs incorporating eccentric contractions in older individuals [75]. The relevance of reducing serum F2-isoprostane concentrations lies in their relationship with free-radical-induced tissue damage, which is frequently associated with neurodegenerative diseases [6]. The present study provides valuable evidence that high-RT programs are not only safe but also beneficial in mitigating oxidative stress, even in older adults. Notably, the positive effects of high-RT training were significantly enhanced in individuals who received additional curcumin supplementation. The reduction in F2-isoprostanes exceeded 40% in the high-RT groups supplemented with curcumin, whereas the decrease was slightly below 30% in RT groups receiving a placebo (Figure 4). This difference reached statistical significance, favoring the Max-Cur group over the Aecc-Pla group, further supporting existing evidence on the antioxidative benefits of curcumin supplementation [76]. Therefore, this study suggests that high-RT training is a safe and effective approach for healthy older adults and that exogenous antioxidant supplementation with curcumin can further enhance antioxidant status in this population.

Cognitive function and most health-related quality of life parameters improved equally following both high-RT programs (Table 2, Figure 4). These findings align with studies that reported the benefits of regular RT participation (1–3 days a week during 4–96 weeks) in enhancing cognitive health and quality of life in older adults [77]. One potential mechanism linking RT to improved cognitive function involves the brain, specifically hippocampus volume and enhanced neurotransmission through BDNF [78]. The hippocampus, a key brain region for learning, memory, and plasticity, is also the primary anatomical site of BDNF expression [79]. According to Park et al. [80], exercise may enhance learning through expression and release of BDNF, a growth factor known to promote functional and structural plasticity in the hippocampus. Therefore, it is not surprising that the increased BDNF levels observed following high-RT programs were correlated with an improvement in cognitive functioning in our participants. Moreover, previous research has highlighted the crucial role of BDNF expression in various mental disorders [81], with increased BDNF levels being associated with symptom improvement [82]. However, while participants in the Max Group did not increase their BDNF levels, they still improved in cognitive function. This improved cognitive function could be mediated by other mechanisms associated with RT, including increased mitochondrial function, enhanced neurotransmission through IGF-1, and decreased homocysteine levels, which trigger complex neurobiological processes, e.g., brain plasticity and angio-neurogenesis, leading to functional and structural brain changes that stimulate cognitive function [22]. Consistent with these findings, our study identified moderate yet significant correlations between BDNF levels and all mental and general health dimensions of quality of life following high-RT protocols.

All performance parameters significantly improved following both high-RT protocols (Table 4, Figure 5). These findings are in line with the results of previous studies, which demonstrated that similar or even shorter RT programs (4–36 weeks) can effectively enhance various performance parameters in sedentary older adults [83]. Pairwise comparisons further indicated that both RT programs were equally effective in improving all physical performance variables, irrespective of supplementation status. The exception to this was the 6-Minute Walk Test, where participants in the curcumin supplementation groups exhibited significantly greater improvements compared to the same high-RT groups receiving placebo. Although research on the effects of curcumin supplementation on aerobic capacity in the elderly is limited, some evidence suggests that curcumin enhances muscle oxygenation at the capillary level, potentially by improving muscle oxygen extraction and delivery [84]. A likely explanation for the observed improvements in the 6-Minute Walk Test following high-RT programs could be the increases in knee extension and flexion strength, as these parameters were strongly correlated. We also observed higher improvements in knee flexion strength capacity in the Max-Cur group compared to both Aecc groups. Collectively, our findings support elastic band high-RT training programs as effective interventions for enhancing strength, aerobic capacity, balance, and agility in older adults.

Generally, curcumin supplementation had a beneficial effect on all dependent variables, although these effects did not always reach statistical significance. For instance, regarding neuro-oxidative markers, curcumin supplementation helped maintain BDNF levels in the control group, whereas participants receiving a placebo experienced a decline. In line with this, previous studies have reported the neuroprotective role of curcumin, particularly in neurodegenerative diseases, suggesting that it can regulate BDNF and the phosphatidylinositol 3 kinase (PI3k)/protein kinase B (Akt) signaling pathways [85]. Our study also confirmed curcumin’s well-established beneficial role in reducing oxidative stress, preventing the interaction between oxygen and nitric oxide to form peroxynitrite [86]. Specifically, our results indicated that, when combined with the Max RT program, curcumin led to a greater reduction in F2-isoprostanes levels compared to Aecc-Pla. Additionally, it helped maintain oxidative stress levels in the control group, whereas they increased in the placebo group. When combined with a high-RT training protocol, curcumin significantly improved performance in the 6 min walk test compared to the corresponding placebo exercise groups, supporting the hypothesis that curcumin supplementation may enhance muscle oxygenation at the capillary level by enhancing nitric oxide availability and, therefore, increasing muscle tissue perfusion and oxygen delivery [84]. Finally, the C-Cur group maintained or slightly improved their overall perception of quality of life, whereas the C-Pla group experienced declines. The differences between the C-Cur and C-Pla groups were statistically significant in terms of general health and social functioning. These findings suggest that curcumin supplementation can be an effective strategy for enhancing quality of life, even in the absence of regular RT programs. Therefore, curcumin supplementation, whether in combination with high-RT or alone, appears to be beneficial for improving various neuro-oxidative markers, physical performance variables, and aspects of general and mental health.

The findings of our study highlight the safety and efficacy of high-RT programs with elastic bands, which in combination with curcumin supplementation further enhance their benefits for several health-related parameters. However, several aspects remain to be explored in future research. Firstly, our study focused on healthy older adults in a medium-term follow-up, while future studies could explore whether similar benefits could be extended to populations with pathologies or chronic conditions, broadening the applicability of these findings. They could also examine longer training durations and include larger sample sizes. Secondly, we did not measure lactate levels following RT programs, which could have provided valuable insight into the potential mediating role of lactate in BDNF expression. Assessing lactate could help clarify whether the observed differences between the Aecc and Max RT programs, particularly the greater effectiveness of Aecc in increasing BDNF levels, were due to variations in lactate production during exercise or other molecular mechanisms (e.g., IGF-1, homocysteine) associated with each type of contraction. Lastly, future studies could assess whether other nutritional supplements could yield greater benefits than curcumin when combined with high-RT protocols, potentially optimizing neuro-oxidative and physical performance outcomes.

## 5. Conclusions

Overall, both high-RT programs were equally effective in producing significant improvements across neuro-oxidative markers, cognitive function, quality of life, and physical performance variables. The only notable exception was BDNF levels, which increased significantly more in the Aecc groups compared to the Max groups. This difference may be attributed to the longer time under tension in Aecc RT sessions, which potentially led to greater lactate accumulation, which is considered a key mediator linking RT with enhanced BDNF expression. The curcumin-based formulation used in this study was shown to be a valuable adjunct to high-RT protocols, enhancing neuro-oxidative markers and physical performance. Additionally, curcumin supplementation alone was effective in preventing BDNF decline, mitigating oxidative stress increases, and improving perceptions of social functioning and general health in the control groups. The moderate and significant correlations between pre–post changes in dependent variables obtained in our study suggest that high-RT training had a global impact on overall well-being and quality of life. Importantly, more than half of the participants in the high-RT groups achieved clinically meaningful improvements in 11 out of 14 dependent variables, highlighting the real-world benefits of these interventions. Collectively, these findings demonstrate that high-RT elastic band training is a safe, effective, and practical strategy for improving neuro-oxidative health, cognitive function, and physical performance in healthy sedentary older adults, with curcumin supplementation serving as a valuable adjunct to further enhance these benefits.

As a practical application, it can be suggested that professionals working with older adults, including those at risk of neurodegenerative disorders, may consider implementing high-RT programs with elastic bands, expecting improvements in neuro-oxidative status, cognition, functionality, and quality of life. Additionally, institutions and policymakers could promote these training modalities to enhance public health.

## Figures and Tables

**Table 1:** Intervention effects on brain-derived neurotrophic factor and F2-isoprostanes.

Variable	Group	Mean ± SD (Pre-Test)	Mean ± SD (Post-Test)	Δ%	*p*-Value (Time)	ES (Time)	Comparison (Group)	*p*-Value (Group)	ES (Group)
Brain-derived neurotrophic factor (pg/mL)	(1) Aecc-Cur	38,738 ± 7834	40,881 ± 6523	5.5	**0.006**	0.29	1−5	<0.001	0.28
							1−6	0.017	0.59
	(2) Aecc-Pla	39,222 ± 8742	41,296 ± 8936	5.3	**0.020**	0.23	2−5	<0.001	0.21
							2−6	0.025	0.46
	(3) Max-Cur	43,737 ± 9565	44,828 ± 8377	2.5	0.256	0.12	3−5	<0.001	0.21
							3−6	0.026	0.07
	(4) Max-Pla	47,309 ± 9208	48,341 ± 8019	2.2	0.282	0.12	4−5	0.001	0.61
	(5) C-Pla	46,016 ± 9587	43,052 ± 9042	−6.4	**<0.001**	0.32			
	(6) C-Cur	46,685 ± 8268	45,284 ± 8590	−3.0	0.075	0.17			
F2-Isoprostanes (pg/mL)	(1) Aecc-Cur	4486 ± 1744	2664 ± 1243	−40.6	**0.003**	1.20	1−5	<0.001	1.83
							1−6	<0.001	1.82
	(2) Aecc-Pla	4497 ± 1647	3185 ± 1027	−29.2	**0.007**	0.95	2−3	0.006	0.97
							2−5	<0.001	1.52
	(3) Max-Cur	3640 ± 1784	2039 ± 1350	−44.0	**0.007**	10.01	2−6	<0.001	1.52
							3−5	<0.001	2.35
	(4) Max-Pla	4694 ± 1937	3313 ± 1878	−29.4	**0.017**	0.72	3−6	<0.001	2.21
							4−5	0.006	1.02
	(5) C-Pla	3858 ± 1292	4738 ± 1012	22.8	**<0.001**	0.75	4−6	0.019	1.08
		
	(6) C-Cur	4539 ± 940	5032 ± 1353	10.9	0.078	0.42			

Notes for the table: Significant differences are highlighted in bold font. SD: standard deviation; %Δ: pre-to-post-intervention percentage change; ES: effect size for the differences (Hedges’ g); Aecc-Cur: accentuated eccentric training + curcumin supplementation group; Aecc-Pla: accentuated eccentric training + placebo group; Max-Cur: maximum strength training + curcumin supplementation group; Max-Pla: maximum strength training + placebo group; C-Pla: passive control + placebo group; C-Cur: passive control + curcumin supplementation group.

**Table 2:** Intervention effects on the cognitive functioning parameters.

Variable	Group	Mean ± SD (Pre-Test)	Mean ± SD (Post-Test)	Δ%	*p*-Value (Time)	ES (Time)	Comparison (Group)	*p*-Value (Group)	ES (Group)
Trail Making Test A (seconds)	(1) Aecc-Cur	47.9 ± 11.7	35.9 ± 13.0	−25.03	**0.001**	0.88	1–5	0.000	1.46
							1–6	0.001	0.03
	(2) Aecc-Pla	39.3 ± 9.1	30.6 ± 9.0	−22.11	**0.002**	0.91	2–5	0.000	3.24
							2–6	0.003	1.31
	(3) Max-Cur	52.4 ± 18.1	32.5 ± 6.8	−37.98	**0.005**	2.66	3–5	0.000	2.77
							3–6	0.000	0.85
	(4) Max-Pla	39.1 ± 8.3	29.4 ± 6.7	−24.81	**0.005**	1.33	4–5	0.000	3.61
							4–6	0.002	1.62
	(5) C-Pla	42.5 ± 13.3	43.1 ± 12.4	1.25	0.928	0.04			
	(6) C-Cur	35.9 ± 12.1	36.0 ± 12.9	0.25	0.395	0.01			
Trail Making Test B (seconds)	(1) Aecc-Cur	98.4 ± 32.2	69.6 ± 21.2	−29.23	**0.003**	1.27	1–5	0.026	1.21
							1–6	0.024	1.13
	(2) Aecc-Pla	82.1 ± 21.2	68.9 ± 18.6	−15.99	**0.001**	0.67	2–5	0.030	0.62
							2–6	0.037	0.50
	(3) Max-Cur	94.2 ± 28.7	68.2 ± 15.7	−27.60	**0.007**	1.51	3–5	0.043	1.17
							3–6	0.035	1.10
	(4) Max-Pla	90.4 ± 25.9	69.5 ± 12.7	−23.12	**0.007**	1.50	4–5	0.030	0.95
							4–6	0.024	0.87
	(5) C-Pla	94.5 ± 30.7	96.2 ± 27.2	1.78	0.065	0.06			
	(6) C-Cur	87.8 ± 17.2	86.1 ± 25.4	1.90	0.656	0.07			

Notes for the table: Significant differences are highlighted in bold font. SD: standard deviation; %Δ: pre-to-post-intervention percentage of change; ES: effect size for the differences (Hedges’ g); Aecc-Cur: accentuated eccentric training + curcumin supplementation group; Aecc-Pla: accentuated eccentric training + placebo group; Max-Cur: maximum strength training + curcumin supplementation group; Max-Pla: maximum strength training + placebo group; C-Pla: passive control + placebo group; C-Cur: passive control + curcumin supplementation group.

**Table 3:** Intervention effects on the health-related quality of life dimensions.

Variable	Group	Mean ± SD (Pre-Test)	Mean ± SD (Post-Test)	Δ%	*p*-Value (Time)	ES (Time)	Comparison (Group)	*p*-Value (Group)	ES (Group)
General health (score)	(1) Aecc-Cur	70.6 ± 11.8	87.8 ± 6.0	24.3	**<0.001**	1.59	1–5	<0.001	0.27
							1–6	<0.001	0.20
	(2) Aecc-Pla	75.0 ± 12.7	86.9 ± 6.9	15.9	**0.002**	1.01	2–5	<0.001	0.53
							2–6	0.004	0.16
	(3) Max-Cur	73.5 ± 12.7	89.0 ± 4.6	21.1	**0.007**	1.41	3–5	<0.001	0.44
							3–6	0.003	0.05
	(4) Max-Pla	75.5 ± 13.4	88.0 ± 7.9	16.6	**0.005**	1.00	4–5	<0.001	0.55
							4–6	0.004	0.20
	(5) C-Pla	66.2 ± 18.6	65.0 ± 17.1	−1.8	0.194	0.06	5–6	0.050	0.43
	(6) C-Cur	73.0 ± 10.5	76.3 ± 9.2	4.6	0.490	0.30			
Social functioning (score)	(1) Aecc-Cur	91.1 ± 8.8	96.5 ± 7.0	5.9	**0.010**	0.58	1–5	<0.001	0.24
							2–5	<0.001	0.63
	(2) Aecc-Pla	95.3 ± 8.0	100.0 ± 0.0	4.9	**0.050**	0.72	3–5	0.013	0.20
							5–6	0.049	0.02
	(3) Max-Cur	90.5 ± 9.5	100.0 ± 8.8	10.5	**0.003**	0.90			
	(4) Max-Pla	86.4 ± 18.1	92.6 ± 10.5	7.2	0.066	0.37			
	(5) C-Pla	88.3 ± 12.8	84.8 ± 11.3	−4.0	**0.040**	0.26			
	(6) C-Cur	88.4 ± 21.4	87.2 ± 17.3	−1.3	0.170	0.05			
Vitality (score)	(1) Aecc-Cur	67.8 ± 12.6	80.0 ± 7.3	18.0	**<0.001**	1.03	1–5	0.012	0.10
							1–6	0.013	0.05
	(2) Aecc-Pla	73.4 ± 14.3	83.5 ± 10.1	13.6	**<0.001**	0.70	2–5	0.002	0.47
							2–6	0.003	0.37
	(3) Max-Cur	64.0 ± 18.8	79.3 ± 13.3	23.9	**<0.001**	0.82	3–5	0.038	0.14
							3–6	0.039	0.15
	(4) Max-Pla	71.0 ± 21.2	86.3 ± 9.7	21.6	**<0.001**	0.82	4–5	<0.001	0.24
							4–6	<0.001	0.20
	(5) C-Pla	66.5 ± 14.3	64.9 ± 14.2	−2.3	**0.038**	0.09			
	(6) C-Cur	67.0 ± 19.0	68.3 ± 18.2	2.0	0.556	0.06			
Mental health (score)	(1) Aecc-Cur	78.3 ± 11.5	86.5 ± 8.4	10.5	**0.001**	0.71	1–5	0.003	0.13
							2–5	<0.001	0.54
	(2) Aecc-Pla	83.1 ± 10.5	88.6 ± 8.8	6.7	**0.018**	0.50	3–5	0.008	0.05
							4–5	0.009	0.14
	(3) Max-Cur	77.1 ± 12.1	86.7 ± 7.3	12.5	**0.017**	0.84			
	(4) Max-Pla	78.4 ± 11.2	84.4 ± 6.4	7.7	**0.042**	0.57			
	(5) C-Pla	76.7 ± 12.1	74.8 ± 11.5	−2.5	0.114	0.14			
	(6) C-Cur	77.6 ± 15.8	78.1 ± 17.0	0.7	0.480	0.03			

Notes for the table: Significant differences are highlighted in bold font. SD: standard deviation; %Δ: pre-to-post-intervention percentage of change; ES: effect size for the differences (Hedges’ g); Aecc-Cur: accentuated eccentric training + curcumin supplementation group; Aecc-Pla: accentuated eccentric training + placebo group; Max-Cur: maximum strength training + curcumin supplementation group; Max-Pla: maximum strength training + placebo group; C-Pla: passive control + placebo group; C-Cur: passive control + curcumin supplementation group.

**Table 4:** Intervention effects on the physical performance parameters.

Variable	Group	Mean ± SD (Pre-Test)	Mean ± SD (Post-Test)	Δ%	*p*-Value (Time)	ES (Time)	Comparison (Group)	*p*-Value (Group)	ES (Group)
30-Second Chair Stand (repetitions)	(1) Aecc-Cur	15.6 ± 3.7	19.8 ± 3.4	26.4	**<0.001**	1.12	1–5	<0.001	1.09
							1–6	<0.001	1.43
	(2) Aecc-Pla	16.2 ± 3.7	18.8 ± 3.3	15.7	**0.001**	0.69	2–5	0.006	0.70
							2–6	<0.001	0.95
	(3) Max-Cur	15.0 ± 2.8	19.4 ± 2.8	29.3	**0.004**	1.48	3–5	0.004	1.18
							3–6	<0.001	1.77
	(4) Max-Pla	13.9 ± 3.0	17.1 ± 2.9	23.0	**0.005**	1.01	4–5	0.039	0.87
							4–6	0.016	1.28
	(5) C-Pla	15.3 ± 4.8	15.1 ± 4.3	−1.7	0.248	0.05			
	(6) C-Cur	15.9 ± 2.4	15.6 ± 2.4	−1.5	0.563	0.10			
Timed Up And Go Test (seconds)	(1) Aecc-Cur	5.4 ± 0.7	4.7 ± 0.5	−13.5	**<0.001**	1.26	1–5	<0.001	0.96
							1–6	<0.001	1.06
	(2) Aecc-Pla	5.6 ± 0.6	5.0 ± 0.6	−11.1	**0.001**	0.99	2–5	0.005	0.77
							2–6	0.041	0.62
	(3) Max-Cur	5.5 ± 0.4	4.8 ± 0.4	−13.6	**0.005**	1.90	3–5	<0.001	0.92
							3–6	<0.001	1.02
	(4) Max-Pla	5.6 ± 0.9	5.0 ± 0.7	−11.5	**0.005**	0.73	4–5	0.008	0.74
							4–6	0.031	0.80
	(5) C-Pla	5.9 ± 1.0	6.0 ± 1.0	1.0	0.272	0.07			
	(6) C-Cur	5.4 ± 0.9	5.5 ± 0.9	1.7	0.265	0.11			
Six-Minute Walk Test (meters)	(1) Aecc-Cur	605 ± 62	661 ± 36	9.2	**<0.001**	1.06	1–2	0.002	0.46
							1–4	0.003	0.50
	(2) Aecc-Pla	597 ± 71	628 ± 69	5.1	**0.001**	0.42	1–5	0.000	0.70
							1–6	0.000	0.83
	(3) Max-Cur	575 ± 69	628 ± 72	9.2	**0.005**	0.70	2–5	0.004	0.35
							2–6	0.000	0.40
	(4) Max-Pla	597 ± 67	627 ± 66	5.1	**0.005**	0.42	3–4	0.036	0.41
							3–5	0.000	0.56
	(5) C-Pla	549 ± 108	545 ± 110	−0.7	0.136	0.03	3–6	0.000	0.64
							4–5	0.013	0.34
	(6) C-Cur	599 ± 98	595 ± 94	−0.7	0.112	0.04	4–6	0.000	0.39
							5–6	0.002	0.08
Manual dynamometry (kg)	(1) Aecc-Cur	30.1 ± 9.3	34.9 ± 9.2	16.1	**<0.001**	0.51	1–5	0.034	0.80
							1–6	0.021	0.82
	(2) Aecc-Pla	32.8 ± 8.2	36.5 ± 8.4	11.1	**0.001**	0.42	2–5	0.009	1.02
							2–6	0.010	1.07
	(3) Max-Cur	28.2 ± 8.4	34.8 ± 8.8	23.2	**0.005**	0.71	3–5	0.050	0.84
	(4) Max-Pla	28.8 ± 9.8	33.6 ± 10.1	16.7	**0.005**	0.46			
	(5) C-Pla	28.4 ± 7.3	27.9 ± 7.4	−1.8	0.130	0.07			
	(6) C-Cur	28.5 ± 8.0	27.6 ± 8.4	−3.2	0.444	0.12			
Knee flexion 60° /s (N·m)	(1) Aecc-Cur	48.3 ± 15.5	78.7 ± 17.8	63.03	**<0.001**	1.82	1–3	0.047	0.54
							1–5	<0.001	1.42
	(2) Aecc-Pla	49.1 ± 14.8	72.3 ± 12.3	47.31	**0.001**	1.71	1–6	<0.001	1.43
							2–3	0.018	1.23
	(3) Max-Cur	49.4 ± 14.3	86.6 ± 10.5	75.40	**0.005**	2.97	2–5	0.001	1.25
							2–6	0.007	1.25
	(4) Max-Pla	52.5 ± 24.3	84.2 ± 24.0	60.30	**0.005**	1.31	3–5	<0.001	2.06
							3–6	<0.001	2.06
	(5) C-Pla	50.7 ± 18.4	48.8 ± 17.5	−3.63	**0.026**	0.01	4–5	<0.001	1.47
							4–6	<0.001	1.47
	(6) C-Cur	52.0 ± 22.3	51.0 ± 21.0	−1.98	0.139	0.05			
Knee extension 60° /s (N·m)	(1) Aecc-Cur	82.6 ± 22.4	127.4 ± 22.4	54.34	**<0.001**	1.95	1–5	0.004	1.18
							1–6	0.038	0.99
	(2) Aecc -Pla	93.1 ± 27.4	130.9 ± 27.4	40.61	**0.001**	1.33	2–5	0.005	1.19
							2–6	0.029	1.00
	(3) Max-Cur	80.8 ± 28.1	126.7 ± 26.4	56.85	**0.005**	1.61	3–5	0.013	1.05
							3–6	0.044	0.87
	(4) Max-Pla	90.9 ± 41.2	129.9 ± 42.5	42.96	**0.005**	0.90	4–5	0.005	0.99
							4–6	0.031	0.82
	(5) C-Pla	91.1 ± 39.0	88.7 ± 39.1	−2.56	0.338	0.07			
	(6) C-Cur	96.1 ± 42.5	95.5 ± 39.1	−0.60	0.826	0.04			

Notes for the table: Significant differences are highlighted in bold font. SD: standard deviation; %Δ: pre-to-post-intervention percentage of change; ES: effect size for the differences (Hedges’ g); Aecc-Cur: accentuated eccentric training + curcumin supplementation group; Aecc-Pla: accentuated eccentric training + placebo group; Max-Cur: maximum strength training + curcumin supplementation group; Max-Pla: maximum strength training + placebo group; C-Pla: passive control + placebo group; C-Cur: passive control + curcumin supplementation group.

**Table 5:** Percentage of participants who exceeded clinically relevant change endpoint/cutoff points of dependent variables.

Variable	Endpoint ^/Cutoff Point *	Benefits/Risks	Aecc-Cur (*n* = 16)	Aecc-Pla (*n* = 13)	Max-Cur (*n* = 10)	Max-Pla (*n* = 10)	C-Cur (*n* = 15)	C-Pla (*n* = 17)
BDNF	<20–30 ng/mL [60] *	Relevant clinical relationship between lower scores and brain shrinkage (8% by age 60)	100%	100%	100%	100%	92%	94%
F2-isoprostanes	12.98 pg/mL decrease [61,62,63] ^	High values raise coronary calcification risk by 23% and acute syndrome risk by 42%	56%	38%	44%	40%	0%	0%
TMT-A	11.70 s decrease [64] ^	High values are associated with a 9% higher prevalence of Alzheimer’s disease	44%	39%	60%	40%	0%	0%
TMT-B	24.40 s decrease [64] ^		44%	15%	40%	40%	6%	6%
30 s Chair Stand Test	≤8 repetitions [65] *	Relevant clinical changes in the appearance of sarcopenia	100%	100%	100%	100%	100%	88%
Timed-Up and Go Test	1.7 s decrease [66,67] ^	Each second decrease reduces fall risk by 9%	50%	46%	60%	50%	0%	0%
6-min Walk Test	28 m increase [67] ^	Relevant changes in the prevention of falls	69%	46%	100%	60%	6%	0%
Grip strength	1 kg increase [68] ^	Decrease in mortality (from 3 to 10%)	100%	100%	100%	100%	0%	0%
Knee extension 60° /s	<94.50 Nm ♂ * <62.30 Nm ♀ [69] *	Relevant clinical changes in the appearance of leg muscle weakness and slow walking speed	100%	100%	100%	100%	66%	53%
Knee flexion 60° /s	<47.00 Nm ♂ * <36.00 Nm ♀ [70] *		100%	100%	100%	100%	60%	58%
General Health	<55.90 points [43] *	Relevant clinical relationship between lower scores and risk of death (SF-36 mental scores: estimates of mortality at 8 years are 7.8%; SF-36 physical scores: estimates of mortality at 8 years are 15.4%)	100%	100%	100%	100%	100%	65%
Social Functioning	<79.20 points [43] *		100%	100%	95%	90%	86%	59%
Vitality	<60.50 points [43] *		100%	100%	100%	90%	73%	70%
Mental Health	<68.30 points [43] *		100%	100%	100%	100%	88%	80%

Notes for the table: The symbol “^” indicates that the values represent an endpoint (i.e., specific levels of improvement associated with positive clinical effects) and “*” represent cutoff points (i.e., thresholds to identify risks or classify participants); BDNF: brain-derived neurotrophic factor; TMT: Trail Making Test.

## Data Availability

The data utilized in this study are part of a broader ongoing project. Access to these data may be granted upon a reasonable request to the corresponding author.

## Abbreviations

The following abbreviations are used in this manuscript:

Aecc	Accentuated eccentric
ANCOVA	Analysis of covariance
ANOVA	Analysis of variance
BDNF	Brain-derived neurotrophic factor
C	Control
CLSI	Clinical and laboratory standards institute
Cur	Curcumin
ELISA	Enzyme-linked immunosorbent assay
ES	Effect size
IGF-1	Insulin-like growth factor 1
LSD	Least significant difference
Max	Maximal strength
MCID	Minimum clinically important difference
METS	Metabolic equivalents
PGF2α	Prostaglandin F2 alpha
Pla	Placebo
RPE	Rating of perceived exertion
RPM	Revolutions per minute
RT	Resistance training
SD	Standard deviations
SF 36	36-Item short-form survey
SST	Serum-separating tube
TMT	Trail Making Test
1RM	One-repetition maximum
